# Effectiveness of a low dose testosterone undecanoate to improve sexual function in postmenopausal women

**DOI:** 10.1186/s12905-015-0270-6

**Published:** 2015-12-02

**Authors:** Reuthairat Tungmunsakulchai, Sukanya Chaikittisilpa, Thiti Snabboon, Krasean Panyakhamlerd, Unnop Jaisamrarn, Nimit Taechakraichana

**Affiliations:** Menopause Research Unit, Reproductive Medicine Division, Department of Obstetrics and Gynecology, Faculty of Medicine, Chulalongkorn University, Rama IV Road, Bangkok, 10330 Thailand; Endocrinology and Metabolism Unit, Department of Internal Medicine, Faculty of Medicine, Chulalongkorn University, Rama IV Road, Bangkok, 10330 Thailand

**Keywords:** Postmenopausal women, Testosterone, Female sexual dysfunction

## Abstract

**Background:**

Adding testosterone to hormonal therapy could improve sexual function and general well-being among women during climacteric. We evaluated the effectiveness of testosterone undecanoate on sexual function in postmenopausal women utilizing the standardized questionnaire FSFI score.

**Methods:**

Postmenopausal women with sexual complaints and Female Sexual Function Index (FSFI) ≤ 26.5 were enrolled in to this randomized, double-blinded, placebo-controlled trial. Participants were randomly assigned to 8-week treatment with either oral testosterone undecanoate 40 mg or placebo twice weekly with daily oral estrogen. The FSFI scores before and after treatment were compared to assess any improvement of sexual function.

**Results:**

Seventy women were recruited of which each group had 35 participants. The baseline characteristics and baseline FSFI scores were comparable between both groups. After 8 weeks of treatment, the FSFI scores significantly improved in both groups when compared to the baseline but the FSFI scores from the testosterone group were significantly higher than in the placebo group post-treatment (28.6 ± 3.6, 25.3 ± 6.7, respectively, p = 0.04). There was no difference in adverse effect between the two groups

**Conclusions:**

The twice weekly addition of testosterone undecanoate to daily oral estrogen was associated with a significant improvement in sexual function among postmenopausal women than the use of the estrogen alone.

**Trial registration:**

ClinicalTrials.gov Identifier NCT01724658 (February 17, 2012).

## Background

Among sexually active menopausal women, sexual dysfunction can significantly affect their quality of life. A previous Thai study reported that up to 82 % of sexually active, postmenopausal women had sexual dysfunction. This was based on the overall Female Sexual Function Index (FSFI) score of ≤ 26.5 among women who had a positive attitude towards sex [1].

Several studies found that adding testosterone to hormonal therapy could improve sexual function and general well-being among women during climacteric [2–12]. Significant improvement was seen by some variables studies when surgical menopausal women were administered 40 mg oral testosterone undecanoate daily together with their estrogen therapy comparable to estrogen alone [13].

For several decades, testosterone undecanoate has been used for the treatment of male hypogonadism. Based on a theoretical assumption that only 6 % of a testosterone dose in male (testosterone undecanoate 120 mg/day) is sufficient for testosterone supplement for females which would correspond to approximately 50 mg per week [14]. However, testosterone undecanoate 50 mg was not available in Thailand when we did the pilot study so we used a single dose of 40 mg capsule and assessed the level of total testosterone and free androgen index after 72 h. The serum levels of total and free testosterone were higher than the baseline levels (total testosterone: treatment VS baseline, 0.7 nmol/l VS 0.5 nmol/l; free androgen index; treatment VS baseline, 1.24 % VS 0.77 %). Based on these preliminary results, we decided to use testosterone undecanoate 40 mg twice weekly to ensure an adequate blood level and minimize androgenic side effects.

The FSFI is a standardized questionnaire used to assess sexual function among postmenopausal women. This questionnaire has been validated in women with clinical female sexual arousal disorder, hypoactive sexual desire disorder, female orgasmic disorder, dyspareunia / vaginismus, multiple sexual dysfunctions and is anonymous patient-based self-reported instrument [15–18]. The FSFI has been used worldwide to evaluate the treatment outcome in clinical trials. We used the Thai version which translation has been validated with high reliability coefficients and internal consistency (*r* = 0.79-0.86, Cronbach’s alpha value = 0.82, FSFI was translated in Thai version and experts agree on validity, The FSFI was tested reliability among Thai postmenopausal women). We then evaluated the effectiveness of testosterone undecanoate on sexual function in postmenopausal women utilizing the standardized questionnaire FSFI score.

## Methods

**Fig. 1:**
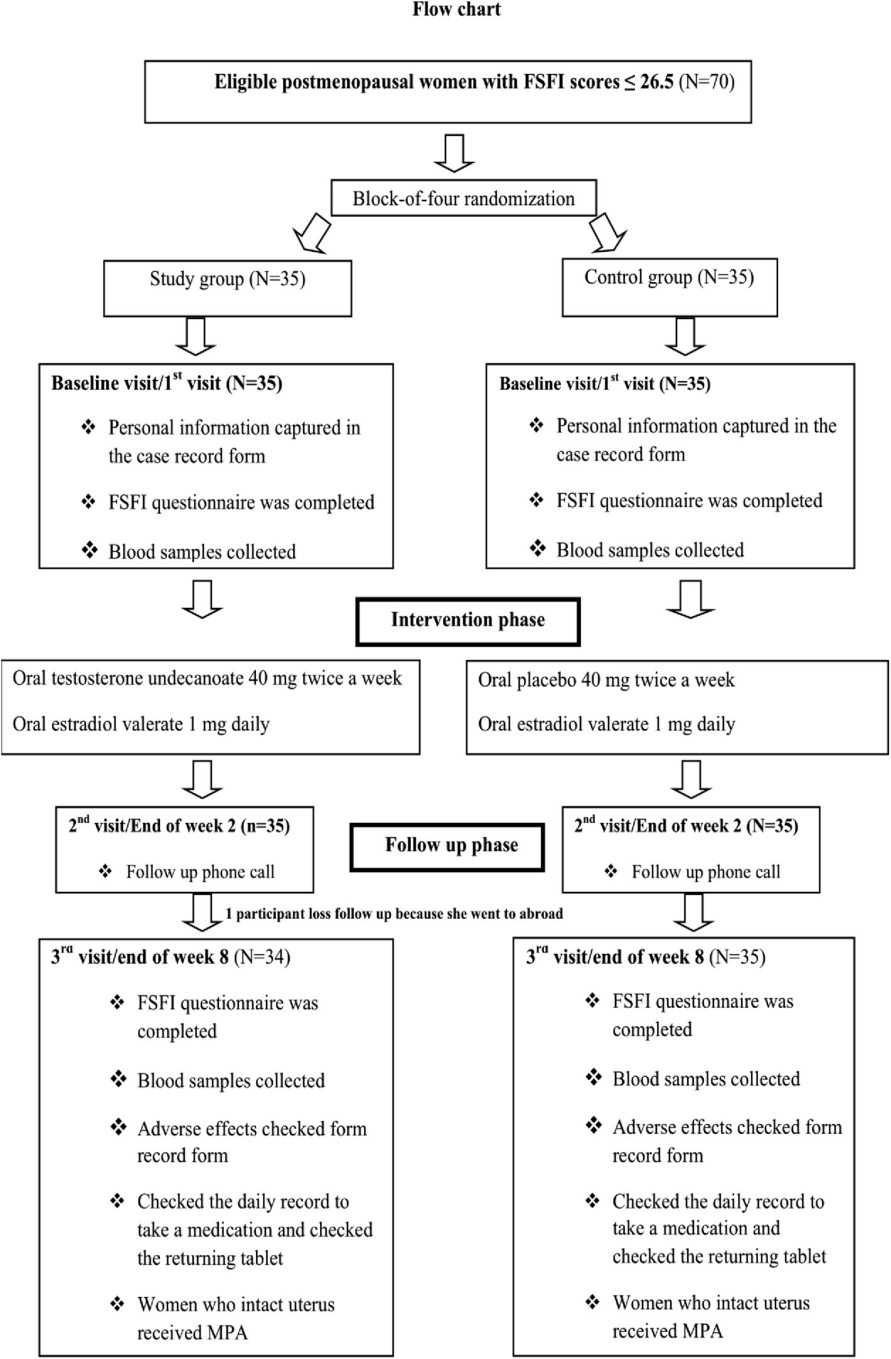
Flow diagram of study, based on CONSORT guidelines 2010

This randomized, double-blind, controlled trial was performed at the menopause clinic, King Chulalongkorn Memorial Hospital. This study was approved by the Institutional Review Board of the Faculty of Medicine, Chulalongkorn University (102/55), and is registered in the clinicaltrials.gov registry (ClinicalTrials.gov Identifier NCT 01724658). This study followed CONSORT guidelines 2010 (Fig. 1).

All sexually active postmenopausal women who visited the menopause clinic between June 2012 and February 2013 were invited to participate in this study. Women between ages 40–60 years were eligible if they were in natural or surgical menopause (history of 12 months of amenorrhea or bilateral removal of both ovaries), sexually active, diagnosed with sexual dysfunction by having FSFI scores ≤ 26.5 and literate. Postmenopausal women with severe vasomotor symptoms, history of venous thrombo-embolism, cerebrovascular disease or cardiovascular disease, cancer or liver disease, history of psychiatric disorders, have used hormonal treatment or any psychiatric drugs in the past 3 months, have abnormal liver function test, abnormal lipid profiles (Triglyceride ≥ 150 mg/dl, LDL ≥ 190 mg/dl) or partner with sexual dysfunction were excluded from the study.

All subjects provided written informed consent before any screening procedures were performed. The sample size was estimated based on the results obtained from our pilot study (the mean difference of FSFI was 4.8). A total of 35 participants per group were needed when the level of statistical significance was set at 0.05 % (α-error = 0.05) to yield a power of 80 % (β-error = 0.2). A 10 % estimation of lost to follow-up was also incorporated into the calculation.

Each participant was interviewed in order to obtain information related to sexual behaviors as well as basic patient information such as age, age at which menopause occurred, type of menopause, marital status, weight, height, BMI, frequency and severity of vasomotor symptoms and sexual history. After being informed about the objective, methodology, and possible side effects of treatment, the participants were randomized into 2 groups by blocks of four, into either 8- week treatment with either 40 mg of oral testosterone undecanoate or identical placebo twice weekly (on Tuesday and Friday with dinner meal) with daily estrogen (1 mg of estrogen valerate). One pharmacist dispensed the testosterone undecanoate or placebo according to the blocks of four randomization list. The testosterone undecanoate or placebo were packed in the dark and sealed envelopes and consecutively numbered for each woman according to the randomization schedule. Each participant was assigned a number which corresponded with the administered drugs in the packed envelops. All participants, care providers, research assistants and biostatisticians were blinded to the study drug. Basic characteristics of the participants were obtained through an interview at baseline. The blood count, liver enzymes, lipid profile and testosterone level were also assessed at baseline visit. Total testosterone and SHBG were measured by the enzyme linked immunosorbent assay technique, inter-assay and intra-assay coefficient of variations (CVs) of total testosterone = 3.74 % and 2.24 %; inter-assay and intra-assay CVs of SHBG = 3.46 % and 2.80 %, respectively).

The correct consumption procedures for testosterone undecanoate, estrogen and placebo were given to all participants. Each participant maintained a diary to record any adverse effects of drugs. For any serious conditions, the participants were instructed to call 24 h hotline. All participants were interviewed by phone after 2 weeks of drug consumption to assess the treatment compliance and adverse effects. Participants were advised to come back if they had any adverse effects and were reminded to record their medication intake. Evaluation after treatment was done at week 8 by using the FSFI questionnaire. The blood samples were collected to assess blood counts, liver enzymes, lipid profile and testosterone level. Participants were interviewed and recorded of any adverse effects such as acne, vaginal bleeding and hirsutism. A 10 mg of medroxyprogesterone acetate (MPA) was given to participants with intact uterus at the end of the study for a total of 14 days.

The primary efficacy variable was the improvement in the FSFI score. The secondary outcomes were the adverse effects of the drug (testosterone undecanoate) such as acne, hirsutism and vaginal bleeding. Statistical analysis was performed by using SPSS 17.0 Software. Descriptive statistics [mean (SD) or median (range) and percentage] were used to present the demographic and, baseline data, and measurement outcomes. ANCOVA was used to compare the FSFI scores after treatment between the groups when the data was normally distributed. Mann–Whitney *U* test was used when the data was not normally distributed. Chi-square test and Fisher’s exact test were used to analyze the side effects between both groups. A *p*-value of < 0.05 was considered statistically significant. An intention-to-treat analysis was used for all outcomes.

## Results

A total of 76 postmenopausal women, who complained of sexual problems, were screened for eligibility. Of these women, 70 were included and randomly assigned to the testosterone (twice weekly 40 mg of oral testosterone undecanoate plus daily 1 mg of oral estradiol valerate) or the placebo group (placebo plus estrogen). Six women were excludes because five women had anti-psychotic drug and one woman had a partner that suffering for erectile dysfunction. Only one woman in the testosterone group was lost to follow up when she relocated to another country. Baseline characteristics between both groups were comparable for age, BMI, duration of menopause, menopausal status and total FSFI score (Table 1).

**Table 1:** Participants’ characteristics

	Testosterone *N* = 35	Placebo *N* = 35	*p* value
Age (years)	53.8 ± 3.6	52.7 ± 4.1	0.23
Body Mass Index (kg/m^2^)	23.4 ± 2.9	23.7 ± 3.2	0.67
Years since menopause	6.2 ± 4.4	5.0 ± 3.4	0.60

Data are presented as mean ± SD, *FSFI* female sexual function index

After 8 weeks of treatment, both groups showed significant improvement in total FSFI score compared to baseline. The total FSFI score (Table 2) and arousal score (Table 3) from the testosterone group were significantly higher than the placebo group (*p* < 0.05).

**Table 2:** FSFI scores at baseline and after 8 weeks of treatment

	Testosterone *N* = 35	Placebo *N* = 35	*p* value^a^
Pre-treatment FSFI score	18.5 ± 5.8	16.3 ± 7.0	0.17
Post-treatment FSFI score	28.6 ± 3.6	25.3 ± 6.7	0.04

Data are presented as mean ± SD; FSFI = Female Sexual Function Index

^a^ANCOVA was used to compare the FSFI scores between groups for post-treatment FSFI

**Table 3:** Effects of testosterone and placebo on various FSFI domains

FSFI domain	Testosterone *N* = 35		Placebo *N* = 35		*p* value^b^
	Baseline	At 8 weeks	Baseline	At 8 weeks	
Desire	2.42	3.47	2.35	3.15	0.99
Arousal	2.73	4.17	2.27	3.45	0.02
Lubrication	3.27	5.08	2.80	4.64	0.28
Orgasm	3.30	4.55	2.75	4.34	0.85
Satisfaction	3.82	5.17	3.35	4.82	0.17
Pain	3.03	5.36	2.85	4.88	0.16

Data are presented as mean

^b^ANCOVA was used to compare the FSFI scores between post-treatment groups

There was no serious adverse effect in this study. The participants reported having mild acne and mild facial hair growth which were comparable between both groups.

No vaginal bleeding was reported in both groups (Table 4). There was no change in hematocrit, liver enzymes or lipid profile during the study period (Table 5). The free androgen index in testosterone group was significant higher than placebo group. (Table 6)

**Table 4:** Adverse effects

Symptoms	Testosterone *N* = 35	Placebo *N* = 35	*p* value
Acne N (%)	6 (17.6)	5 (14.2)	0.10
Hirsutism N (%)	3 (8.8)	3 (8.5)	0.70

**Table 5:** Serum blood count, liver enzymes and lipid profiles before and after treatment

		Testosterone *N* = 35	Placebo *N* = 35	*p* value
HCT	Before	40.1 ± 3.52	40.6 ± 2.44	0.45
mg%	After	40.6 ± 3.21	40.9 ± 3.00	0.65
SGOT	Before	23.2 ± 6.20	21.5 ± 5.83	0.78
U/l	After	23.6 ± 5.00	21.9 ± 5.01	0.74
SGPT	Before	22.5 ± 10.25	22.7 ± 13.01	0.94
U/l	After	19.5 ± 9.71	19.9 ± 13.27	0.89
TC	Before	219.6 ± 29.97	220.1 ± 30.07	0.94
mg/dl	After	216.8 ± 28.56	214.9 ± 38.26	0.81
HDL	Before	59.7 ± 10.36	62.3 ± 12.76	0.34
mg/dl	After	58.8 ± 10.80	62.8 ± 13.45	0.17
TG	Before	90.1 ± 29.22	93.8 ± 37.58	0.65
mg/dl	After	101.6 ± 36.29	110.9 ± 51.29	0.39
LDL	Before	142.8 ± 27.17	139.1 ± 27.36	0.58
mg/dl	After	137.5 ± 27.92	129.9 ± 31.92	0.29

*HCT* hematocrit, *SGOT* serum glutamic-oxaloacetic transaminase, *SGPT* seum glutamic-pyruvic transaminase, *TC* total cholesterol, *HDL* high-density lipoprotein, *TG* triglyceride, *LDL* low-density lipoprotein

Data are presented as mean ± SD

**Table 6:** Total serum testosterone and free androgen index

Hormone level	Testosterone group *N* = 35	Placebo group *N* = 35	*p* value
Total T			
Baseline	0.47 ± 0.30	0.46 ± 0.26	P = 0.89
Post-treatment	0.82 ± 0.58	0.48 ± 0.31	P < 0.05
FAI			
Baseline	0.89 ± 0.98	0.78 ± 0.64	P = 0.85
Post-treatment	1.42 ± 1.54	0.51 ± 0.35	P < 0.05
SHBG			
Baseline	81.09 ± 31.45	107.60 ± 45.84	P < 0.05
Post-treatment	65.84 ± 26.40	87.52 ± 64.51	P = 0.83

Total T, total testosterone nmol/ml; FAI, free androgen index

Data are presented as mean ± SD

## Discussion

We found that the 8 weeks of testosterone undecanoate 40 mg given twice weekly with daily estrogen was associated with a significant improvement in the women’s overall sexual function when compared with estrogen alone, especially for the arousal domain. The recorded adverse effects of low dose testosterone undecanoate administered for 8 weeks was comparable to the placebo group for acne and hirsutism. We did not observe any vaginal bleeding. There were no changes in hematocrit, liver enzymes or lipid profile after 8-weeks of treatment. However, it is possible that the sample size used in this study may not have been powered enough to detect differences between the two groups with respect to the safety variables.

Among couples, sexual intercourse is one of the most important factors that can significantly affect the status and health of the relationship. Female sexual dysfunction is the result of interplay of biological, psychological, physiological, status/health of the relationship and sociocultural factors. However, in postmenopausal women, hormonal factor is deemed to be an important factor influencing female sexual dysfunction. Estrogen has been shown to be associated with the woman’s sexual drive/desire and used widely to relieve menopausal symptoms, vaginal dryness, dyspareunia and to improve libido. On the contrary, the role of testosterone in postmenopausal hormone therapy is less clear. It is not as commonly prescribed as estrogen which may partly be due to the concern over side effect and long-term safety.

Previous studies have shown that low levels of testosterone were significantly associated with reduced sexual desire, arousal and responsiveness in women [2, 3]. Other studies showed that by adding testosterone to hormonal therapy it can improve sexual function in postmenopausal women [2–10]. These data indicate that supplemental testosterone may help increase sexual function in postmenopausal women but its short- and long-term safety remains to be of concern [19]. Our study was aimed to look into the short-term effects of adding testosterone undecanoate to estrogen for the improvement of female sexual dysfunction among postmenopausal women as well as its possible androgenic side effects when administered a low dose of 40 mg twice weekly.

Sexual function is difficult to assess because it is composed of a combination of biological and psychological factors/variables. Protocols to assess quality of sexual life varied from study to study and make it difficult to compare outcomes. In the present study, we used the FSFI questionnaire which has been validated for various aspects of sexual function and performance in pre and postmenopausal women on estrogen and testosterone. FSFI is a well-standardized questionnaire which objectively assesses the subjective outcome. We used the FSFI total score of 26.5 to be the optimal cutoff score because it has been demonstrated to differentiate women with and without sexual dysfunction [18].

## Conclusions

This study appears to support the addition of the lower dose and twice weekly administration of testosterone undecanoate to daily estrogen for postmenopausal women with female sexual dysfunction, however, a larger study with a longer duration is needed to confirm the long-term efficacy and safety profiles. Besides the monitoring of vaginal bleeding, endometrial assessment should be properly evaluated to confirm the testosterone effect on endometrial safety. Partner’s influence should also be taken into account because it is known to have high influence over female’s sexuality.

## References

[1] Peeyananjarassri K, Liabsuetrakul T, Soonthornpun K, Choobun T, Manopsilp P (2008). Sexual functioning in postmenopausal women not taking hormone therapy in the Gynecological and Menopause Clinic, Songklanagarind Hospital measured by female sexual function index questionaire. J Med Assoc Thai.

[2] Schwenkhagen A, Studd J (2009). Role of testosterone in the treatment of hypoactive sexual desire disorder. Maturitas.

[3] Anita HC (2010). The pathophysiology of hypoactive sexual desire disorder in women. Inter J Gynecol Obstet.

[4] Shifren JL, Glenn DB, James AS, Peter RC, John EB, Geoffrey PR (2000). Transdermal testosterone treatment in women with impaired sexual function after oophorectomy. N Engl J Med.

[5] The North American Menopause Society (2005). The Role of Testosterone Therapy in Postmenopausal Women: Position Statement of The North American Menopause Society.

[6] Shifren JL, Davis SR, Moreau M, Waldbaum A, Bouchard C, DeRogatis L (2006). Testosterone patch for the treatment of hypoactive sexual desire disorder in naturally menopausal women: results from the INTIMATE NM1 Study. Menopause.

[7] Somboonporn W, Bell RJ, Davis SR (2006). Androgen and menopause. Curr Opin Obstet Gynecol.

[8] Kingsberg S, Shifren JL, Wekselman K, Rodenberg C, Koochaki P, DeRogatis L (2007). Evaluation of the clinical relevance of benefits associated with transdermal testosterone in postmenopausal women with hypoactive sexual desire disorder. J Sex Med.

[9] Somboonporn W, Bell JR, Davis RS, Seif MW, Bell R (2005). Testosterone for peri and postmenopausal women. Cochrane Database Syst Rev.

[10] Alvaro M, Brenda J, Jeremy PW, Mark L (1997). Testosterone supplementation for hypogonadal impotence: Assessment of biochemical measures and therapeutic outcomes. J Urol.

[11] Köhn FM, Schill WB (2003). A new oral testosterone undecanoate formulation. World J Urol.

[12] Flöter A, Carlström K, Schoultz B, Nathorst-Böös J (2000). Administration of testosterone undecanoate in postmenopausal women: effects on androgens, estradiol, and gonadotrophins. Menopause.

[13] Flöter A, Nathorst-Böös J, Carlström K, Schoultz B (2002). Addition of testosterone to estrogen replacement therapy in oophorectomized women: effects on sexuality and well-being. Climacteric.

[14] Miller K, Biller K, Beauregard C, Lipman G, Jones J, Schoenfeld D (2006). Effects of testosterone replacement in androgen deficient women with hypopituitarism: a randomized, double-blind, placebo-controlled study. J Clin Endocrino Metab.

[15] Rosen R, Brown C, Heiman J, Leiblum S, Meston C, Shabsigh R (2000). The female sexual function index (FSFI): a multidimensional self-report instrument for the assessment of female sexual function. J Sex Marital Ther.

[16] Meston CM (2003). Validation of the female sexual function index (FSFI) in women with female orgasmic disorder and in women with hypoactive sexual desire disorder. J Sex Marital Ther.

[17] Meston CM, Derogatis LR (2002). Validated instruments for assessing female sexual function. J Sex Marital Ther.

[18] Wiegel M, Meston CM, Rosen R (2005). The female sexual function index (FSFI): cross-validation and development of clinical cutoff scores. J Sex Marital Ther.

[19] Hong Z, Lena S, Britt M, Elina E, Angelica L (2006). Effects of testosterone treatment on endometrial proliferation in postmenopausal women. J Clin Endocrino Metab.

